# Retraction: Smart rose flower like bioceramic/metal oxide dual layer coating with enhanced anti-bacterial, anti-cancer, anti-corrosive and biocompatible properties for improved orthopedic applications

**DOI:** 10.1039/d3ra90026f

**Published:** 2023-03-28

**Authors:** N. Murugan, L. Kavitha, E. Shinyjoy, D. Rajeswari, K. Vimala, S. Kannan, D. Gopi

**Affiliations:** a Department of Chemistry, Periyar University Salem 636011 Tamilnadu India dhanaraj_gopi@yahoo.com +91 427 2345124 +91 427 2345766; b Centre for Nanoscience and Nanotechnology, Periyar University Salem 636011 Tamilnadu India; c Department of Physics, School of Basic and Applied Sciences, Central University of Tamilnadu Thiruvarur 610 101 Tamilnadu India; d Department of Physics, Periyar University Salem 636 011 Tamilnadu India; e Proteomics and Molecular Cell Physiology Laboratory, Department of Zoology, Periyar University Salem 636011 Tamilnadu India

## Abstract

Retraction of ‘Smart rose flower like bioceramic/metal oxide dual layer coating with enhanced anti-bacterial, anti-cancer, anti-corrosive and biocompatible properties for improved orthopedic applications’ by N. Murugan *et al.*, *RSC Adv.*, 2015, **5**, 85831–85844, https://doi.org/10.1039/C5RA17747B.

The Royal Society of Chemistry, with the agreement of the named authors, hereby wholly retracts this *RSC Advances* article due to concerns with the reliability of the data in the published article.

There are unexpected similarities between the FTIR spectra in Fig. 1a, b and d, which represent different samples.

The HRSEM image in Fig. 3c represents a scaled version of part of the image presented in Fig. 3b. However, both images are presented with the same scale bar and represent different experimental conditions.

The authors informed the Editor that the characterization of the original samples was outsourced, and they do not have the original raw data for the published results.

Given the significance of the concerns about the validity of the data, and the lack of raw data, the findings presented in this paper are not reliable.

N. Murugan and K. Vimala were contacted but did not respond. S. Kannan responded but did not confirm whether they agreed to retract the article.

Signed: L. Kavitha, E. Shinyjoy, D. Rajeswari and D. Gopi

Date: 16th March 2023

Retraction endorsed by Laura Fisher, Executive Editor, *RSC Advances*

## Supplementary Material

